# Negatively Charged Composite Nanofibrous Hydrogel Membranes for High-Performance Protein Adsorption

**DOI:** 10.3390/nano12193500

**Published:** 2022-10-06

**Authors:** Qiuxia Fu, Dandan Xie, Jianlong Ge, Wei Zhang, Haoru Shan

**Affiliations:** 1 School of Textile and Clothing, Nantong University, Nantong 226019, China; 2 National and Local Joint Engineering Research Center of Technical Fiber Composites for Safety and Health, Nantong University, Nantong 226019, China

**Keywords:** electrospun nanofibers, functional hydrogel layers, composite membranes, electronegativity, protein adsorption

## Abstract

Nanofibrous materials are considered as promising candidates for fabricating high-efficiency chromatography media, which are urgently needed in protein pharmaceuticals purification and biological research, yet still face several bottlenecks. Herein, novel negatively charged composite nanofibrous hydrogel membranes (NHMs) are obtained by a facile combination of electrospinning and surface coating modification. The resulting NHMs exhibit controllable morphologies and chemical structures. Benefitting from the combined effect of the stable framework of silicon dioxide (SiO_2_) nanofiber and the function layer of negatively charged hydrogel, as well as good pore connectivity among nanofibers, NHMs exhibit a high protein adsorption capacity of around 1000 mg g^−1^, and are superior to the commercial cellulose fibrous adsorbent (Sartobind^®^) and the reported nanofibrous membranous adsorbents. Moreover, due to their relatively stable physicochemical and mechanical properties, NHMs possess comprehensive adsorption performance, favorable resistance to acid and solvents, good selectivity, and excellent regenerability. The designed NHMs composite adsorbents are expected to supply a new protein chromatography platform for effective protein purification in biopharmaceuticals and biochemical reagents.

## 1. Introduction

With the continuous development of the biopharmaceutical industry, the improvement of highly effective bioseparation technology, especially protein separation and purification techniques, has become increasingly important and thus has attracted growing attention [1,2]. Generally, industrial protein separation and purification are implemented by liquid chromatography based on chromatographic columns. Compared with the increasingly mature chromatography operation method, adsorption and separation media, as the key components of the chromatographic column, greatly affected the efficiency, separation accuracy, energy consumption, and cost of the downstream process of protein products [3,4]. Therefore, the design and development of highly effective chromatography media are urgently required.

Traditionally, gel microbead media (20–200 μm) that possess rich nanosized pores are most commonly used to pack chromatographic columns [5]. However, due to their relatively large swelling property and poor mechanical strength, these gel beads are inclined to deform and then accumulate more tightly with each other under high driving pressure and liquid flow rate, causing an increase in flow resistance and energy consumption and a decline of processing rate and throughput [6,7]. The inorganic particle with large rigidness was introduced as the substrate to prepare the chromatography media to solve the above-mentioned problem [8,9]. Although the deformation problem of the microbead media has been effectively mitigated, the close accumulation problem under external force still exists. Differing from microbead materials, nanofibrous materials have been deemed the most promising alternative substrates of high-performance chromatography media, attributed to their fascinating properties, including rich pore structure, good pore connectivity, large specific surface area, and ease of functional modification [10,11].

In comparison to various fabrication methods of nanofibers, including stretching, template synthesis, melt-blown, flash spinning, and islands-in-a-sea, electrospinning has drawn great attention for the design and preparation of protein adsorbents owing to its wide range of raw materials, the controllable structure of single fibers and aggregates, as well as good technical associativity [12,13,14]. Electrospun nanofibrous protein adsorption media are mainly prepared via the following methods. The most commonly used method is surface-modifying the nanofibrous membranes with functional agents, such as DEAE-surface-functionalized cellulose nanofiber [15], cellulose diacetate nitrate nanofiber [16], cellulose-graft-polypropionic acid nanofibers [17], carboxyl-group-functionalized ethylene-vinyl alcohol and silk nanofibers [18,19]. In addition, a variety of nanofibrous adsorbents were fabricated by introducing functional agents into nanofibers via the blend electrospinning method, which was followed by in situ modification treatment involving in situ carboxylated ethylene-vinyl alcohol and polyvinyl alcohol (PVA) nanofibers [20] and sodium alginate/PVA composited nanofibers [21]. Additionally, some composite nanofibrous adsorbents were fabricated by directly doping functional polymers or inorganic particles into nanofibers without further modification; for instance, hydroxyapatite nanoparticles decorated cellulose triacetate nanofibers [22], zinc-doped hydroxyapatite and nylon 6 composite nanofibers [23], and nanoparticle-doped polystyrene/polyacrylonitrile composite nanofibers [24]. Nevertheless, most of the current electrospun nanofibrous protein adsorbents were fabricated by modifying the organic polymer nanofibrous substrates (*e.g.,* PVA nanofiber, regenerated cellulose nanofiber), which might face severe swelling problems and the following decline of application performance during long-term use in the liquid environment [25,26]. Fortunately, coating a functional polymer layer on inorganic nanofiber can functionalize the inorganic nanofibrous membranes with efficient protein adsorption capability and endow the composite materials with superior swelling resistance, resulting in relatively long-term performance stability. However, as far as we know, no relevant research work has been reported.

In this study, we developed composite nanofibrous hydrogel membranes (NHMs) by subtly combining electrospinning and surface coating modification techniques. Electrospun SiO_2_ nanofibers were taken as the rigid framework, and negatively charged hydrogel of citric acid crosslinked PVA was introduced as the functional layer of NHMs. Morphologies and chemical structures of the resulting NHMs were regulated by controlling the esterification degree and loading of a negatively charged hydrogel. The mechanical properties and pore structure of the resulting NHMs were systematically investigated. Profiting from the composite nanofibrous structure and electronegativity properties of high carboxylation, the optimized NHMs presented a relatively large static equilibrium adsorption capability of about 1000 mg g^−^^1^ and a high dynamic capability of almost 700 mg g^−^^1^. The protein adsorption processes of NHMs obeyed Langmuir isothermal and pseudo-first-order kinetic models. Moreover, the adsorption performance of NHMs could be controlled by regulating pH and the ionic strength of the buffer solution. More importantly, the NHMs exhibited good resistance to acid and solvents, selectivity, and excellent reusability. With the merits of a controllable structure and excellent protein adsorption performance, NHMs might be recognized as a potential alternative to the current chromatography media for high-efficiency protein adsorption.

## 2. Materials and Methods

### 2.1. Materials

PVA (Mw = 88,000), citric acid (CCA), acetic acid, polyphosphoric acid, phosphoric acid (85 wt%), disodium hydrogen phosphate (Na_2_HPO_4_), monosodium orthophosphate (NaH_2_PO_4_), anhydrous sodium sulfate (Na_2_SO_4_), isopropyl alcohol (IPA), alcohol, urea, sodium dodecyl sulfate (SDS), and sodium hydroxide (NaOH) were obtained from Aladdin Reagent Co., Ltd., Shanghai, China. Lysozyme with an isoelectric point (pI) of 10.7 was taken as the main template protein to investigate the protein adsorption performance of the NHMs. Additionally, pepsin (pI = 1), ovalbumin (pI = 4.7), BSA (pI = 4.8), bromelain (pI = 9.5), and papain (pI = 8.75) were selected as the template proteins to evaluate the adsorption selectivity of NHMs. All the template proteins used in the adsorption experiments were supplied by Sangon Biotech Co., Ltd., Shanghai, China. Distilled water used throughout the experiment was supplied by the Heal-Force system. All reagents were directly used without any processing.

### 2.2. Fabrication of NHMs

Firstly, SiO_2_ nanofibrous membranes (NFMs) were fabricated according to our previously reported works [27]. Then, 10 wt% PVA solution was prepared by dissolving PVA powder in distilled water in an airtight container at 70 °C for 6 h. Afterward, a fixed proportion of PVA solution, CCA powder, polyphosphoric acid, and distilled water was blended in a beaker according to the experimental design. During the dipping coating process, several pieces of square SiO_2_ NFMs with a side length of 5 cm were soaked in a 30 mL modification solution. After being slightly shaken for 30 min, the SiO_2_ NFMs adsorbed with modification solutions were extracted from the beaker and laid flat on aluminum foil. Subsequently, the composited SiO_2_ NFMs were dried at 50 °C and transferred into a blast oven at 110 °C. After heat treatment for 1 h, the modified SiO_2_ NFMs were thoroughly washed to neutral using distilled water. Finally, the composite CCA-co-PVA/SiO_2_ nanofibrous hydrogel membranes (NHMs) were obtained after drying.

### 2.3. Measurements of Protein Adsorption Capability

The protein adsorption performance of the negatively charged NHMs was measured by selecting lysozyme with a high isoelectric point (10.7) as the main template protein due to its relatively stable properties. In detail, 0.02 M phosphate-buffered saline (PBS) was prepared using Na_2_HPO_4_, NaH_2_PO_4_, phosphoric acid, and NaOH solution, and the pH value of the PBS was adjusted from 2 to 12. Afterward, protein solutions with various concentrations (0 to 1.2 mg mL^−^^1^) were prepared by dispersing protein powder in PBS. The quantitative adsorption capacity test method was the same as our previous research [28]. Typically, 20 mg NHMs were immersed and shaken vigorously in a 24 mL protein solution. Then, the NHMs were separated from the hybrid system, and the absorbance of the supernate was measured by an ultraviolet-visible spectrophotometer. According to the Lambert–Beer law, the protein concentration in the corresponding solution could be computed, and the adsorption capacity of the NHMs could be quantitatively calculated.

### 2.4. Kinetic and Isothermal Adsorption Process Analysis of the NHMs

Pseudo-first- and second-order models were introduced to quantitatively analyze the protein adsorption kinetics of NHMs, which are, respectively, expressed as Equations (1) and (2) [29,30]:

(1) $$Q_{t}=Q_{e}\left(1-e^{-\frac{k_{1}}{2.303}\cdot t}\right)$$

(2) $$Q_{t}=\frac{k_{2}Q_{e}{}^{2}t}{1+k_{2}Q_{e}t}$$

where *Q_t_* (mg g^−1^) is the adsorption capacity at a given contact time (*t*), *Q_e_* (mg g^−1^) is the saturated adsorption capacity of the NHMs, and *k*_1_ and *k*_2_ are relevant adsorption rate constants of the two models, respectively. Moreover, Langmuir and Freundlich models were utilized to systematically evaluate the isothermal adsorption process of NHMs towards protein, which are, respectively, expressed as Equations (3) and (4) [31,32]:

(3) $$Q_{m}=Q_{e}\frac{\left(1+K_{L}C_{e}\right)}{K_{L}C_{e}}$$

(4) $$\mathrm{lg}Q_{e}=\frac{\mathrm{lg}C_{e}}{n}+\mathrm{lg}K_{F}$$

where *Q_m_* and *Q_e_* represent the maximum adsorption capacity and the adsorption capability under various initial protein concentrations (mg g^−1^), *C_e_* represents the equilibrium protein concentration (mg mL^−1^), 1/*n* represents the adsorption intensity constant, and *K_L_* and *K_F_* represent the Langmuir and Freundlich isotherm constant, respectively.

### 2.5. Measurements of Influence Factors on the Performance of NHMs

Firstly, a certain amount of Na_2_SO_4_ powder (0 to 1.0 M) was added to the lysozyme solution to evaluate the influence of ionic strength on the application properties of NHMs. To research the acid-alkali and solvent resistance, the NHMs were immersed in acid (pH = 2) and alkaline (pH = 10) PBS for 1 to 7 days or soaked in various chemical reagents for a fixed 12 h. Then, the acid-alkali and solvent resistance of the NHMs were judged by testing the performance of the resulting NHMs after being washed to neutral. Reusability of the NHMs was studied by cyclic testing dynamic binding of the NHMs under gravity drive (1 kPa) and using 1 M Na_2_SO_4_ solution as the desorption reagent. Additionally, the detailed methods of adsorption capacity calculation, dynamic adsorbing capability measurement, and selectivity test have been elaborated in the previously reported studies [28].

### 2.6. Instruments and Characterizations

The morphological and chemical structure of the resulting NHMs were characterized, respectively, by employing field emission scanning electron microscopy (FE-SEM, Gemini SEM 300, Carl Zeiss, Oberkochen, Germany) and Fourier transform infrared spectrum (FT-IR, Nicolet IS 50, Thermo Scientific, Waltham, MA, USA). Tensile mechanical properties of wet and dry states of the membranes were measured by taking an XQ-1C tensile tester (Shanghai New Fiber Instrument Co., Ltd., Shanghai, China). The absorbance of the protein solutions (280 nm) was measured by introducing a PG 2000pro UV-vis fiber-optic spectrophotometer (Shanghai Fuxiang Optics Co., Ltd., Shanghai, China). The pH value of the buffer was detected using a PHS-3C pH meter (Shanghai INESA Scientific Instrument Co, Ltd., Shanghai, China). Zeta potentials of the representative NHMs were measured by employing Zeta Sizer Nano-ZS90 (Malvern Instruments Ltd., Great Malvern, UK). CFP-1100AI capillary flow porometer (Porous Materials Inc., Ithaca, NY, USA) was introduced to characterize the pore structure of the original SiO_2_ membranes and the composite NHMs.

## 3. Results and Discussion

**Scheme 1:**
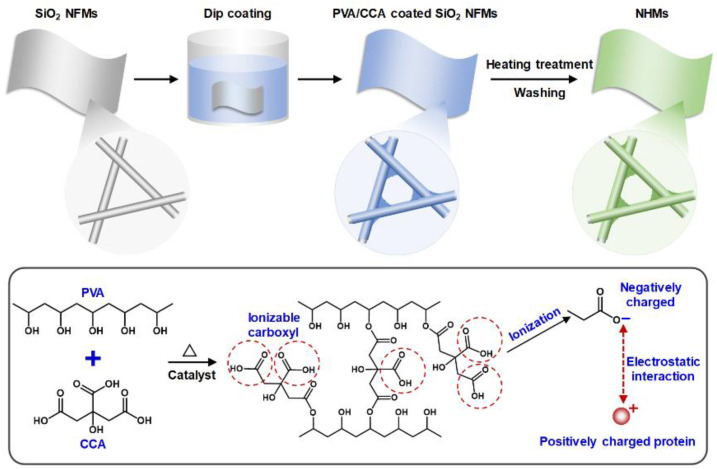
Schematic diagram of the preparation process, chemical reaction process, and protein adsorption mechanism of the NHMs.

This work aims to fabricate high-performance membranous adsorbents for high-efficiency protein and pharmaceutical purification applications. Different from the traditional organic membranous adsorbents, we designed and fabricated composite NHMs according to the following criterion: (1) the membranous materials should be hydrophilic; (2) the adsorbent should graft abundant adsorption groups under a mild and relatively unpolluted modification process; (3) and the materials should possess good resistance to swelling. As demonstrated in Scheme 1, SiO_2_ NFMs with good hydrophilic features and water swelling resistance were chosen as the rigid skeleton. PVA with excellent hydrophily and surface activity was selected as the polymeric coating layer. Environmentally friendly CCA with ionizable carboxylic acid groups was taken as the crosslinking agent and modifier towards the water-soluble PVA. Finally, negatively charged NHMs with protein adsorption capability were obtained.

**Figure 1:**
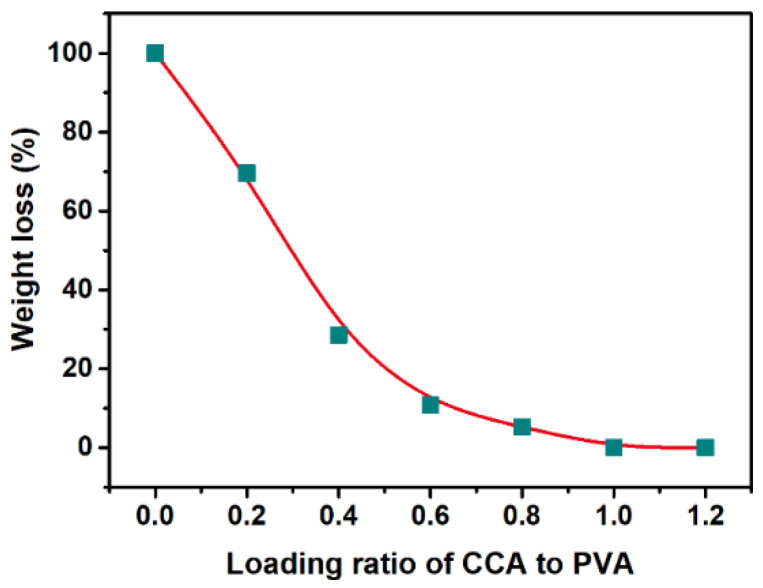
Influences of loading ratio of CCA to PVA on the structural stability of the functional CCA-co-PVA hydrogel coating layer.

**Figure 2:**
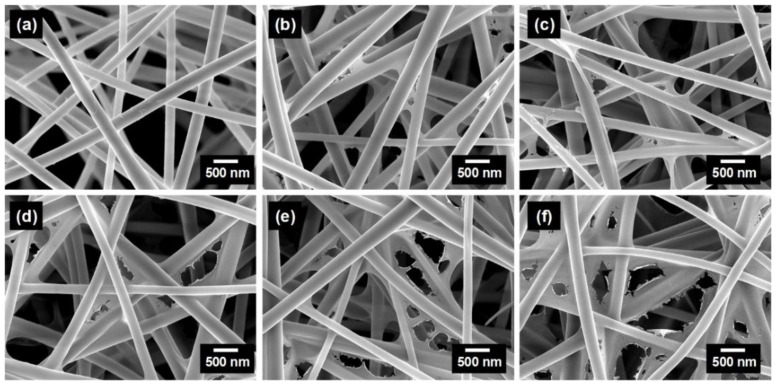
FE-SEM images of (**a**) SiO_2_ NFMs and the composite NHMs prepared from different CCA-to-PVA loading ratios of (**b**) 1:1, (**c**) 2:1, (**d**) 3:1, (**e**) 4:1, and (**f**) 5:1.

Since the composite NHMs are used in aqueous environments, the first target was thus to confirm the minimum loading amount of CCA to fully crosslink PVA, thereby preventing dissolution of PVA during the application process. As presented in Figure 1, after being immersed in water for 24 h, the composite CCA-co-PVA hydrogel film showed a large weight loss (˃20 wt%) as the mass loading ratio of CCA to PVA was less than 0.4. With the increase in CCA loading amount, the weight loss of CCA-co-PVA hydrogel film gradually reduced and reached a stable structural state as the mass loading ratio of CCA to PVA increased to 1.0. On this basis, the influence of CCA-to-PVA loading ratio (at a fixed PVA loading of 1 wt%) on the morphologic structure and adsorption capability of the composite materials were further investigated. As shown in Figure 2, the CCA-to-PVA loading ratio increased from 1:1 to 4:1, and the CCA-co-PVA hydrogel film was slightly covered among SiO_2_ NFMs. After the loading ratio increased to 5:1, CCA-co-PVA hydrogel film among nanofibers could be observed, which could be ascribed to the increase in viscosity of the modification solution (Figure S1).

**Figure 3:**
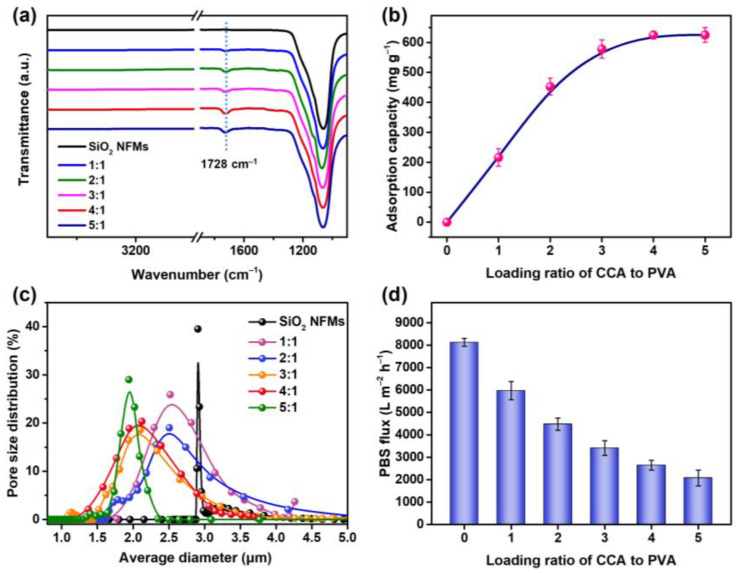
(**a**) FT-IR spectra, (**b**) protein adsorption capacities, (**c**) pore size distribution, and (**d**) PBS flux of the SiO_2_ NFMs and the composite NHMs prepared from different loading ratios of CCA to PVA.

FT-IR spectra, pore size distributions, and adsorption capability of the corresponding nanofibrous materials are presented in Figure 3. Significantly, the corresponding characteristic absorption peak around 1728 cm^−1^ gradually enhanced (Figure 3a), demonstrating the increased crosslinking and carboxylate degree of the functional CCA-co-PVA hydrogel film [33]. In contrast, the static lysozyme adsorption capacity of the NHMs gradually increased to 600 mg g^−1^ as the CCA-to-PVA loading ratio increased to 4:1 and then kept balance (Figure 3b). The differential distribution of hydrogel film also greatly affected the pore structure of the NHMs. As shown in Figure 3c, the initial SiO_2_ NFMs presented a relatively large pore size of mainly around 2.9 μm. The average pore size of NHMs gradually decreased, and less than 2.0 μm at a CCA-to-PVA loading ratio of 5:1. Due to the declined pore size, PBS flux of these membranes also decreased from about 8000 L m^−2^ h^−1^ to 2000 L m^−2^ h^−1^ (Figure 3d). By comprehensively analyzing the above experimental results, the CCA-to-PVA loading ratio of 4:1 was selected to conduct the following experiments.

**Figure 4:**
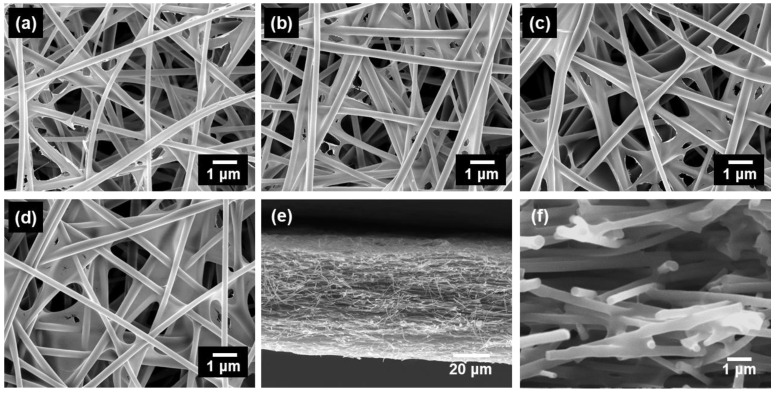
FE-SEM images of composite NHMs with different PVA loading amounts of (**a**) 1.0, (**b**) 1.5, (**c**) 2.0, (**d**) 2.5 wt% under a fixed CCA to PVA content ratio of 4:1. (**e**,**f**) Cross-sectional images of the representative NHMs sample.

Apart from the CCA-to-PVA loading ratio, the PVA loading amount was considered to be another primary factor that greatly affects the structure and performance of the resulting CCA-co-PVA/SiO_2_ hydrogel membranes, which should be systematically investigated. Herein, PVA loading concentration was tailored from 1 wt% to 2.5 wt%. The FE-SEM results (Figure 4a–d) demonstrated that the adhesive structure of the functional hydrogel layer among SiO_2_ nanofibers increased along with increasing PVA loading amount, which could be ascribed to the accordingly increased viscosity (Figure S2). Nevertheless, pores among SiO_2_ nanofibers were almost filled at the PVA loading amount of 2.5 wt%. Moreover, as seen from the cross-sectional image in Figure 4e,f, the NHMs were closely packed, and SiO_2_ nanofibers were bonded to each other, illustrating that the functional CCA-co-PVA hydrogel layer was not only covered on the surface but also filled within the composite membranes.

**Figure 5:**
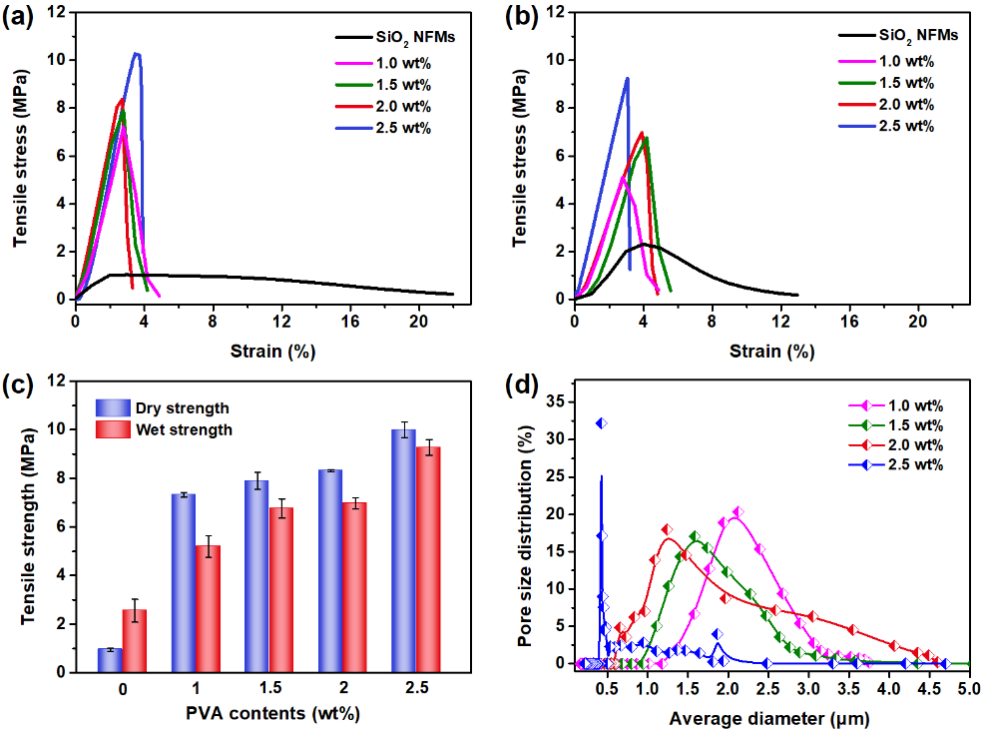
Stress–strain curves of the SiO_2_ NFMs and relevant NHMs in (**a**) dry and (**b**) wet states. (**c**) The corresponding strength and (**d**) pore size distribution of these samples.

Since the composite hydrogel nanofibrous membranes need to bear external force during the practical application, relatively good mechanical properties are thus necessary to satisfy the requirement, especially in chromatographic column filling and terminal use [34]. Thus, the relationship between mechanical property and the PVA loading amount of the NHMs was systematically investigated. Figure 5a,b present the tensile curves of these samples in dry and wet states. Significantly, it was found that, after coating with a functional hydrogel layer, the tensile strength of the composite membranes was gradually enhanced with increasing PVA loading amount both at dry and wet states. The dry SiO_2_ NFMs exhibited a relatively small tensile strength of 0.95 MPa and a large breaking elongation of 22.4%, ascribing to the no-adhesion structure and disordered distribution of SiO_2_ nanofibers. The tensile strength of the composite NHMs rapidly increased to 7.3 MPa, and breaking elongation declined to around 2.0% at 1 wt% PVA loading amount. The stress increased to 10 MPa as the PVA loading concentration reached 2.5 wt%, almost ten times higher than the initial SiO_2_ NFMs. The dramatic increase in tensile strength could be attributed to the enhanced CCA-co-PVA bonding structure among SiO_2_ nanofibers. As seen in Figure 5c, the strength of the wetted NHMs was inferior to that of the dry state after wetting. This result might be attributed to the water-swelling effect of water on the hydrogel bonding points among SiO_2_ nanofibers [35]. Moreover, the average pore diameter of the NHMs also gradually decreased with the increasing PVA loading concentration (Figure 5d).

**Figure 6:**
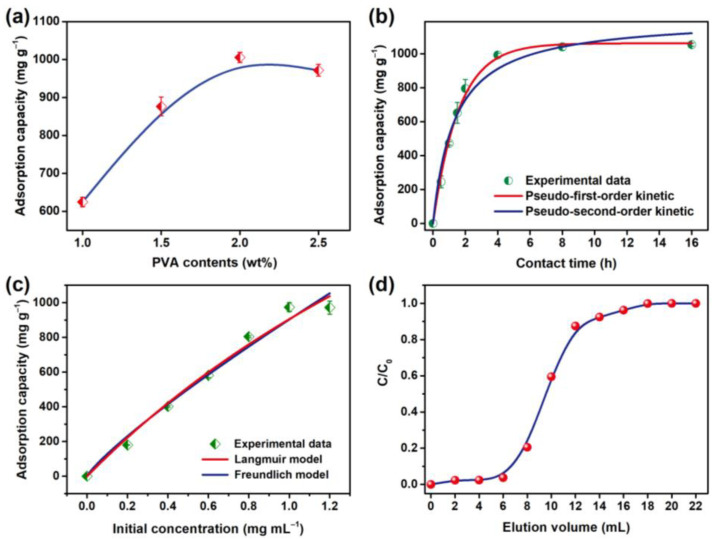
(**a**) Protein adsorption capacities of the NHMs with various PVA contents. Experimental results and the resultant fitting curves related to the (**b**) adsorption isotherm and (**c**) kinetics adsorption curves of CCA-co-PVA/SiO_2_ NFMs with 2 wt% PVA. (**d**) The obtained breakthrough curve of NHMs (3 layers, total thickness of around 0.2 mm).

The influence of PVA loading amount on the adsorption capability of NHMs was further researched. As shown in Figure 6a, under the fixed CCA-to-PVA loading ratio of 4:1, the protein adsorption capability of NHMs gradually improved with increasing PVA loading amount. The maximum adsorption capacity reached almost 1000 mg g^−1^ at 2 wt% PVA loading content. Results of adsorption kinetic are displayed in Figure 6b. Obviously, the NHMs exhibited rapid adsorption kinetic in the initial adsorption time of 2 h and then achieved adsorption equilibrium of around 1000 mg g^−1^ after about 5 h, which is superior to the commercial cellulose fibrous adsorbent (Sartobind^®^) and reported nanofibrous membranous adsorbents [18,25]. The higher protein concentration at the initial adsorption stage provides a larger contact probability for the fast uptake of NHMs towards protein molecules. As the adsorption process continued, adsorption sites were gradually occupied by protein molecules, and then the number of available binding sites declined, thus decreasing the adsorption rate [36]. For investigating the binding mechanism of NHMs, the resulting kinetic data were further analyzed using the pseudo-first-order and pseudo-second-order kinetic models (Equations (1) and (2)) [29,37]. The fitting curves and relevant correlation coefficients (*R*^2^) are displayed in Figure 6b and Table 1. Significantly, the protein adsorption kinetics of NHMs presented a better fit to the pseudo-first-order model, and the fitted equilibrium capacity was 1062 mg g^−1^. This result declared that the adsorption rate was controlled by a physical adsorption mechanism, which was consistent with the designed electrostatic adsorption interaction between NHMs and proteins [30,38].

Figure 6c and Table 2 exhibit the analysis of the isothermal adsorption process of NHMs. The adsorption capacity of NHMs presented rapid growth in the incipient stage and finally reached equilibrium with the increased protein concentration. Quantitative analysis of the isothermal adsorption process was conducted by introducing the classical Langmuir and Freundlich models as expressed in Equations (3) and (4), respectively [31,32]. As can be seen from these results, the adsorption capacity of the NHMs matched better with the Langmuir model (*R*^2^ of 0.982), demonstrating the monolayer adsorption process of NHMs towards protein [39]. The Langmuir theoretical capacity of NHMs was 3846.8 mg g^−1^, illustrating their outstanding application prospect. Moreover, it could be expected that the adsorption capability of the NHMs will be optimized by further controlling the preparation method and the physical and chemical structure.

Considering the significance of dynamic adsorption performance on the application, a dynamic breakthrough experiment of the NHMs was performed under a fixed gravity driving of protein solution (liquid height of 10 cm). As shown in Figure 6d, a thin column of NHMs (three layers, total thickness of about 0.2 mm) exhibited a typical breakthrough curve with a nearly complete adsorption volume of 6 mL. Subsequently, the concentration of the solution flowing through the NHMs column was gradually increased before reaching its initial concentration (1 mg mL^−1^) at a breakthrough volume of approximately 20 mL. Quantitative calculation results demonstrated that the total breakthrough capacity of NHMs could reach nearly 66% of the saturated dynamic capability (696 mg g^−1^), almost 2 times superior to the reported membranous materials [18]. By comprehensively considering the relatively large dynamic adsorption capacity and small driving force of gravity (1.0 kPa), it might be inferred that the NHMs could greatly improve efficiency and reduce energy consumption during real protein separation and purification applications.

**Figure 7:**
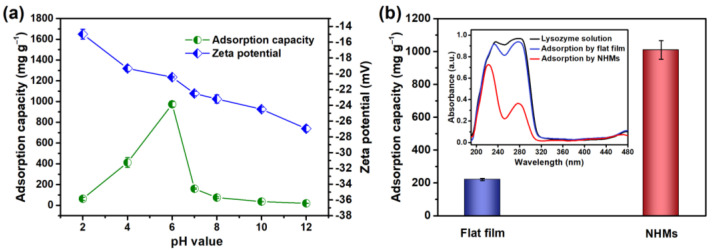
(**a**) Adsorption performance and Zeta potential of the NHMs at various pH values. (**b**) Performance comparison of the NHMs and functional hydrogel flat film; insert exhibits the UV spectra of the lysozyme solution before and after adsorption.

Since the adsorption force of protein on NHMs mainly relied on their electrostatic interaction, and the pH value of the buffer solution greatly affected the surface charges of both protein molecule and membranous adsorbent [40,41], the adsorption capability of NHMs at different pH conditions urgently needed to be investigated. As seen from Figure 7a, with increasing buffer solution pH, the NHMs presented a linear increase and the subsequent decrease in adsorption capability, and the maximum capability was obtained at pH 6. Because the surface charge property of the template protein of lysozyme has been investigated comprehensively [42], the Zeta potential of NHMs was thus systematically measured to further verify the results and interpretation. The NHMs presented a gradually enhanced negative Zeta potential with the increased pH value, resulting in improved surface electronegativity. On the other hand, the positive surface electricity of the template protein decreases with the increasing pH value and then exhibits electronegativity as the pH is larger than the isoelectric point of the lysozyme (10.7) [43]. Thus, the electrostatic interaction forces between NHMs and template protein might reach a maximum, and a relatively large adsorption capability was obtained around a pH of 6. In addition, by comparing the performance of NHMs and CCA-co-PVA flat hydrogel film (Figure 7b), it was found that the capability of the flat hydrogel film (about 220 mg g^−1^) was almost one-fifth of the NHMs, illustrating the great impact of nanofibrous morphologic structure on application properties of the composite materials.

**Figure 8:**
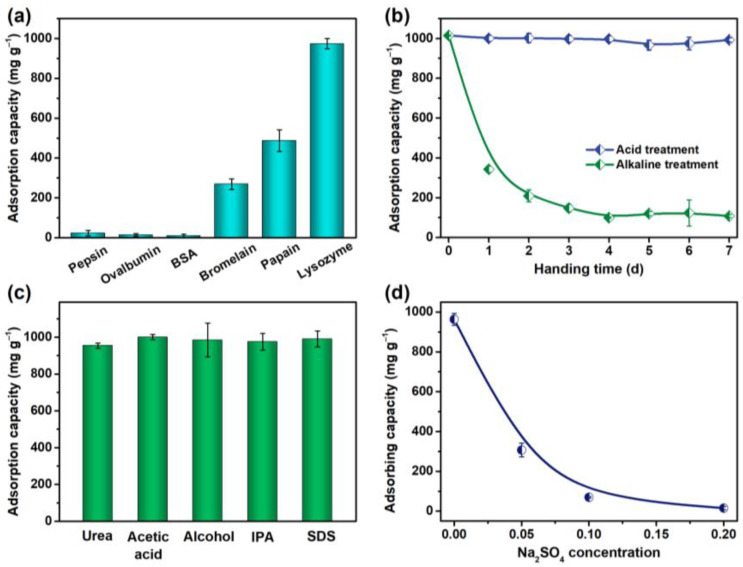
(**a**) Adsorption selectivity and (**b**) adsorption capacity of the NHMs after being exposed to acid (pH = 2) and alkali (pH = 10) buffer for different lengths of time. (**c**) Solvent resistance and (**d**) influence of ionic strength of the NHMs.

To realize protein separation and purification in practical application, the NHMs should possess certain adsorption selectivity. As illustrated in Figure 8a, under a fixed pH of 6, the NHMs presented a different adsorption capability towards various proteins with differential properties (isoelectric point and molecular weight). The almost 0 mg g^−1^ adsorption capacity of NHMs toward pepsin, ovalbumin, and BSA could be ascribed to their surface electronegativity, resulting from their lower isoelectric point than the buffer pH [44]. As mentioned above, the surface electricity of both protein and composite membranes could be adjusted by controlling pH, and the molecular weight also influences the adsorption interaction; therefore, it could be envisaged that the NHMs can realize selective protein adsorption and separation in practical utilization [45]. In addition, the resistance properties of the NHMs against acid, alkaline, and organic solvents were systematically evaluated due to their large influences on the practical utility, especially in the desorption, purification, elution, and regeneration processes. As shown in Figure 8b, the adsorption capacity of NHMs remained relatively stable after being treated with acid buffer throughout the test process, whereas performance largely declined after being immersed in the alkaline buffer. It could be ascribed to the fact that the morphologic and chemical structures of the functional CCA-co-PVA hydrogel layer can keep stable in the acid environment while largely being hydrolyzed and destroyed in the alkaline condition [46]. Furthermore, as shown in Figure 8c, the NHMs presented a relatively stable performance after being treated with various solutions, including urea, acetic acid, alcohol, IPA, and SDS, showing excellent organic solvent resistance.

As a new type of ion-exchange chromatography media, protein elution and material regeneration processes usually need to be conducted under strong ionic strength conditions [47]; therefore, the influence of ionic strength on the performance of NHMs was investigated. Similar to the previous research [28,48], there was a negative correlation between the adsorption capability of NHMs and the ionic strength of the buffer solution (Figure 8d), and the adsorption capacity almost rapidly declined with the increasing Na_2_SO_4_ concentration, demonstrating the easy elution and regeneration properties of a NHMs-packed chromatographic column. Moreover, chromatography media also need beer sterilization treatment to maintain microorganism safety [49]. Herein, sterilization treatment of the NHMs was conducted by heating at 120 °C in a drying oven for 2 h. As exhibited in Figure S3, almost no obvious change in adsorption capacity could be observed throughout the heat treatment sterilization process, highlighting the excellent stabilizability of the NHMs.

**Figure 9:**
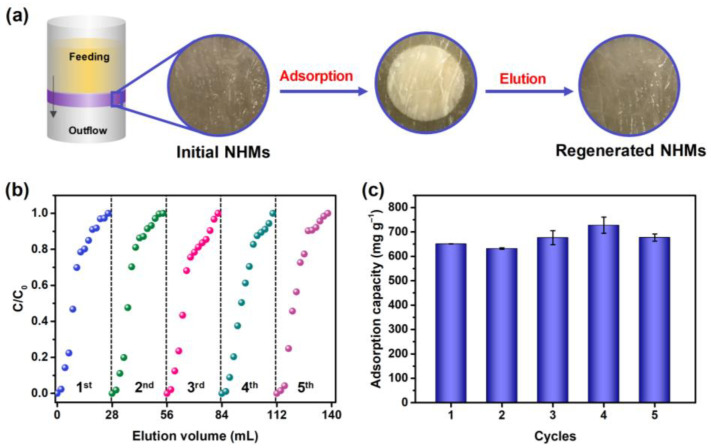
(**a**) Schematic diagram and photograph of the NHMs under different adsorption states during dynamic cycle tests. (**b**) The representative cycling breakthrough curves and (**c**) dynamic adsorption capacity of the thin-layer chromatography column packed with three layers of NHMs in five cycles.

Generally, protein chromatographic column usually needs experience in recycling adsorption, elution, and regeneration processes; therefore, it is necessary to explore the recyclability of the composite nanofibrous hydrogel membranes [8,25]. Herein, the recyclability of the NHMs was evaluated by supervising the saturated dynamic adsorption capability of the NHMs packed chromatographic column. As shown in Figure 9a, the hydrated NHMs-packed-column exhibits translucent and dark yellow. After the dynamic adsorption experiment, an obvious milky white area could be observed within the area of protein solution flow, and then the hydrated composite membranes returned to the initial state after being dynamically washed by 0.2 M Na_2_SO_4_ solution. Corresponding results of the quantitative analysis were recorded in Figure 9b,c; it was obvious that nearly no decline in adsorption performance was observed during the five using cycles, demonstrating the outstanding cycle performance of NHMs.

## 4. Conclusions

In summary, we demonstrated the preparation and investigation of negatively charged composite nanofibrous hydrogel membranes as a new platform for protein purification by combining electrospun nanofiber and functional coating techniques. Due to the synergistic effects of the cooperative core layer of SiO_2_ nanofiber and highly carboxylated functional hydrogel layer, as well as relatively good pore connectivity, the resultant NHMs presented a large protein adsorption capability of around 1000 mg g^−1^, which was realized by taking the CCA-to-PVA loading ratio of 4:1 and the PVA loading concentration of 2 wt% as the optimum fabrication parameters. The adsorption performance of NHMs is superior to the commercial cellulose fibrous adsorbent (Sartobind^®^) and reported nanofibrous membranous adsorbents. Additionally, the adsorption capability of the NHMs could be controlled by regulating the PBS environment, including pH and ionic strength, and the maximum adsorption capacity of NHMs could be achieved under a weak acid PBS environment (pH of 6.0) and without loading of ionic strength. Benefiting from its relatively stable physicochemical and mechanical properties, the NHMs also presented good acid/solvent resistance, selectivity, and regenerability, demonstrating the efficient practicality of protein purification. We believe the present work will pave the way for designing and fabricating the next-generation ion-exchange chromatographic column for protein adsorption and purification in the bio-pharmaceuticals industry.

## Figures and Tables

**Table 1:** The relevant kinetic parameters of lysozyme adsorption on the NHMs.

Pseudo-First-Order Kinetic Model			Pseudo-Second-Order Kinetic Model		
*Q_e_* (mg g^−1^)	*k*_1_ (min^−1^)	*R* ^2^	*Q_e_* (mg g^−1^)	*k*_2_ (min^−1^)	*R* ^2^
1062.0	1.447	0.996	1214.959	0.617	0.972

**Table 2:** The calculated Langmuir and Freundlich constants and correlation coefficients.

Langmuir Model			Freundlich Model		
*Q_m_* (mg g^−1^)	*K_L_* (mL mg^−1^)	*R* ^2^	*K_F_*	1/*n*	*R* ^2^
3846.8	0.307	0.982	902.8	1.180	0.976

## Data Availability

The data used to support the findings of this study are available from the corresponding author on request.

## Supplementary Materials

The following supporting information can be downloaded at: https://www.mdpi.com/article/10.3390/nano12193500/s1, supplementary methods: buffer flux measurement; Figure S1: Viscosity of the coating solution with various loading ratios of CCA to PVA under a fixed PVA concentration of 1 wt%; Figure S2: Viscosity of the coating solutions with various PVA loading amount under a fixed CCA-to-PVA loading ratio of 4:1; Figure S3: Influence of heat sterilization time on the adsorption capacity of NHMs.
