# Supplementary material for: Negatively Charged Composite Nanofibrous Hydrogel Membranes for High-Performance Protein Adsorption
Source: Nanomaterials (Basel). 2022 Oct 6;12(19):3500. doi: 10.3390/nano12193500 (PMC9565482; doi:10.3390/nano12193500)
Supplement: Supplementary file 1 [file nanomaterials-12-03500-s001.zip › nanomaterials-1874132-supplementary.pdf]

nanomaterials

Supplementary Materials

Negatively Charged Composite Nanofibrous Hydrogel Membranes for High-Performance Protein Adsorption

Qiuxia Fu [1,2], Dandan Xie [1], Jianlong Ge [1], Wei Zhang [1,2,*] and Haoru Shan [1,2,*]

[1] School of Textile and Clothing, Nantong University, Nantong 226019, China
[2] National and Local Joint Engineering Research Center of Technical Fiber Composites for Safety and Health, Nantong University, Nantong 226019, China

Buffer Flux Measurement

To measure buffer flux of the NHMs, a piece of NHMs was held between a pair of filter tubes (diameter of about 15 mm). After that, PBS (pH = 6) was continuously injected into the upper glass tube and keep a fixed fluid level of 10 cm. Subsequently, volume (L) of the PBS run through the NHMs in the first minute was recorded. Thus, PBS flux of the NHMs could be calculated according to the following equation:

$$F = \frac{v}{s \cdot t} \tag{1}$$

where F is the PBS flux (L m^{-2} h^{-1}), v represents the average value of PBS volume (L) run through NHMs recorded in the first minute, s represents cross section area of the glass tube (m^2), t is fixed time of 1/60 (h).

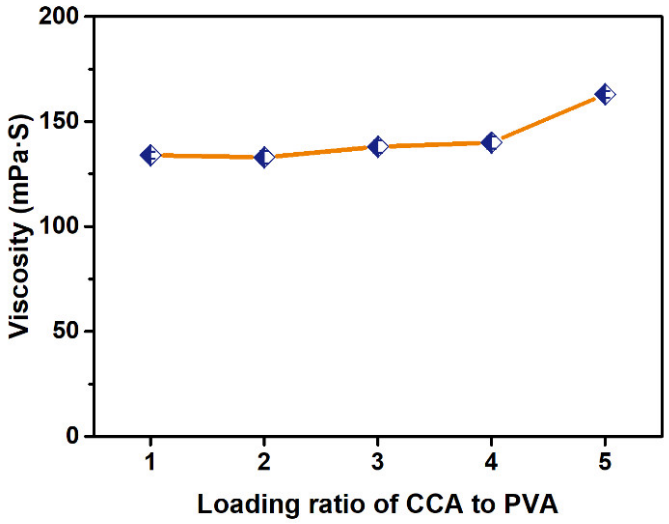

Figure S1. Viscosity of the coating solution with various loading ratios of CCA to PVA under a fixed PVA concentration of 1 wt%.

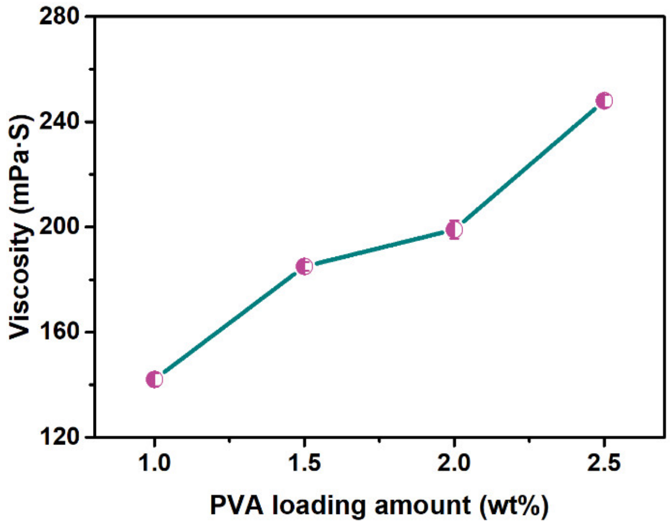

Figure S2. Viscosity of the coating solutions with various PVA loading amount under a fixed CCA to PVA loading ratio of 4:1.

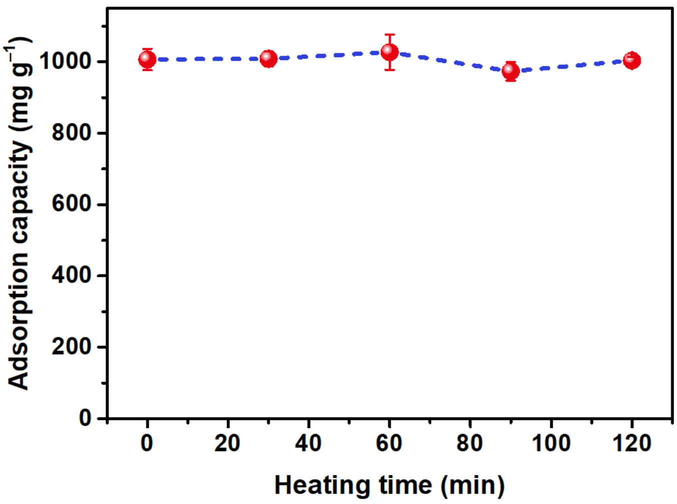

Figure S3. Influence of heat sterilization (120 °C) time on the adsorption capacity of NHMs.
